# Supplementary material for: Pathophysiological Response to SARS-CoV-2 Infection Detected by Infrared Spectroscopy Enables Rapid and Robust Saliva Screening for COVID-19
Source: Biomedicines. 2022 Feb 1;10(2):351. doi: 10.3390/biomedicines10020351 (PMC8962262; doi:10.3390/biomedicines10020351)
Supplement: Supplementary file 1 [file biomedicines-10-00351-s001.zip › biomedicines-1556059-supplementary.pdf]

Supplementary Information

Pathophysiological response to SARS-CoV-2 infection detected by infrared spectroscopy enables rapid and robust saliva screening for COVID-19

Seth T. Kazmer[1], Gunter Hartel[2†], Harley Robinson[1†], Renee S. Richards[1], Kexin Yan[3], Sebastiaan J. Van Hal[4], Raymond Chan[4], Andrew Hind[5], David Bradley[5], Fabian Zieschang[5], Daniel J. Rawle[3], Thuy T. Le[3], David W. Reid[6, 7], Andreas Suhrbier[3,8], Michelle M Hill[1,9*]

Contents

Figure S1. Culture supernatant ATR-FTIR spectra and subtractive analysis of Vero cell SARS-CoV-2 infection model.
Figure S2. Full ATR-FTIR spectra and subtraction analysis of mouse oral lavage.
Figure S3. Significant features of in vivo infection mouse model using LogWorth FDR analysis.
Figure S4. Acceptable technical variance between replicates using pairwise Euclidean distancing analysis of human samples.
Figure S5. Human subject ATR-FTIR close-up of averaged spectra per three groups.
Table S1. Summary of cohorts.

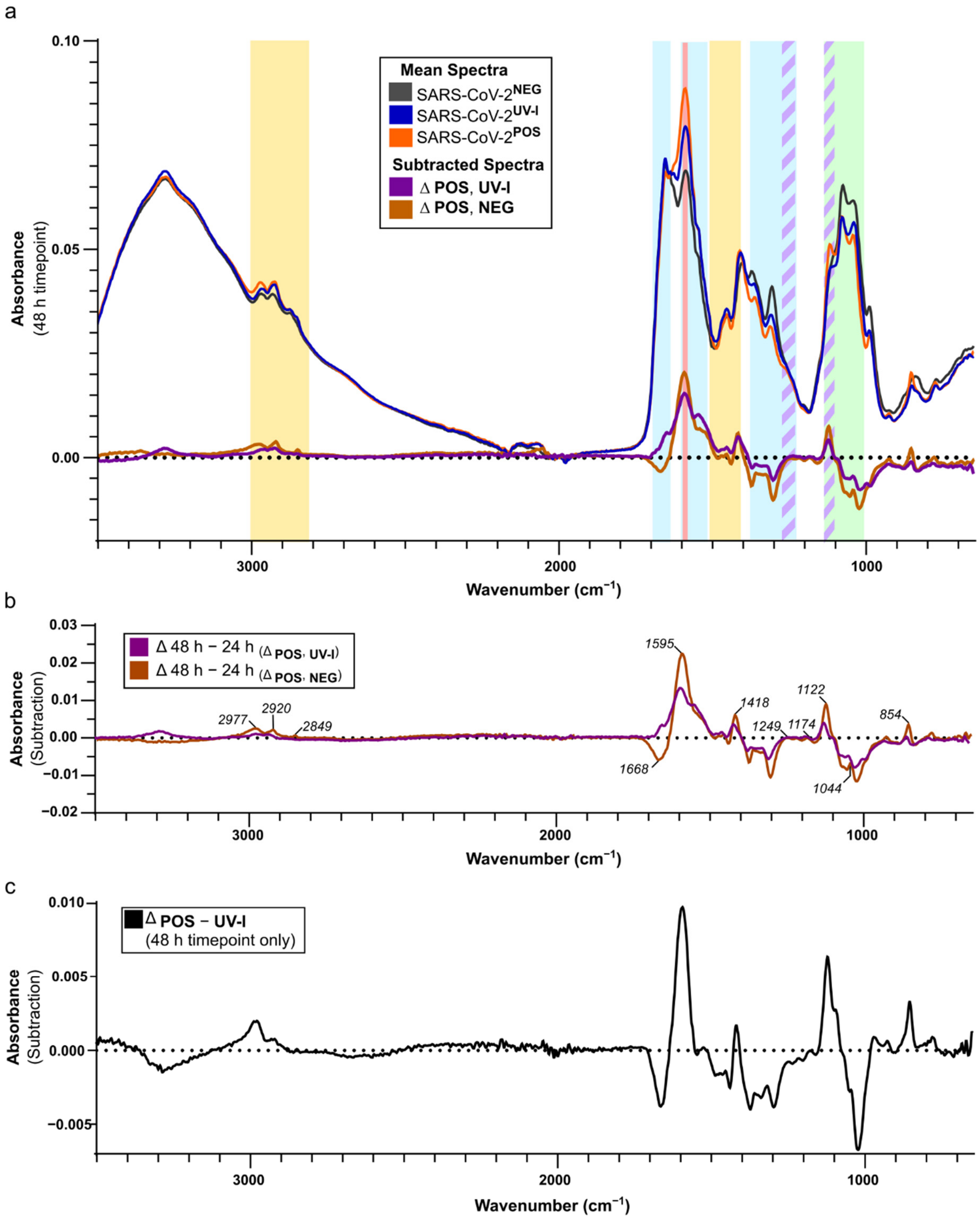

Figure S1. Culture supernatant ATR-FTIR spectra and subtractive analysis of Vero cell SARS-CoV-2 infection model. Refer to Figure 1 for experimental details. **a.** Spectra of all three groups at 48 h time point (grey, blue, orange) with subtracted spectra (Δspectra) along the X-axis, SARS-CoV-2 spectra subtracted from media control (dark orange), SARS-CoV-2 spectra subtracted from inactivated control (purple). Bands signified by colours: Aliphatic (yellow), Amide I/II/III (cyan), severity (red), Saccharide (green), phosphodiester asymmetric stretching ($\nu_{as}PO_2^-$) and symmetric stretching ($\nu_{s}PO_2^-$) (purple stripes). **b.** Isolated subtraction spectra (48 h – 24 h) of ΔSARS-CoV-2 from media control (dark orange) and inactivated control (purple). **c)** The unique spectral changes of active infection at 48 h: spectra subtraction of SARS-CoV-2 from Inactivated SARS-CoV-2 at 48 hr.

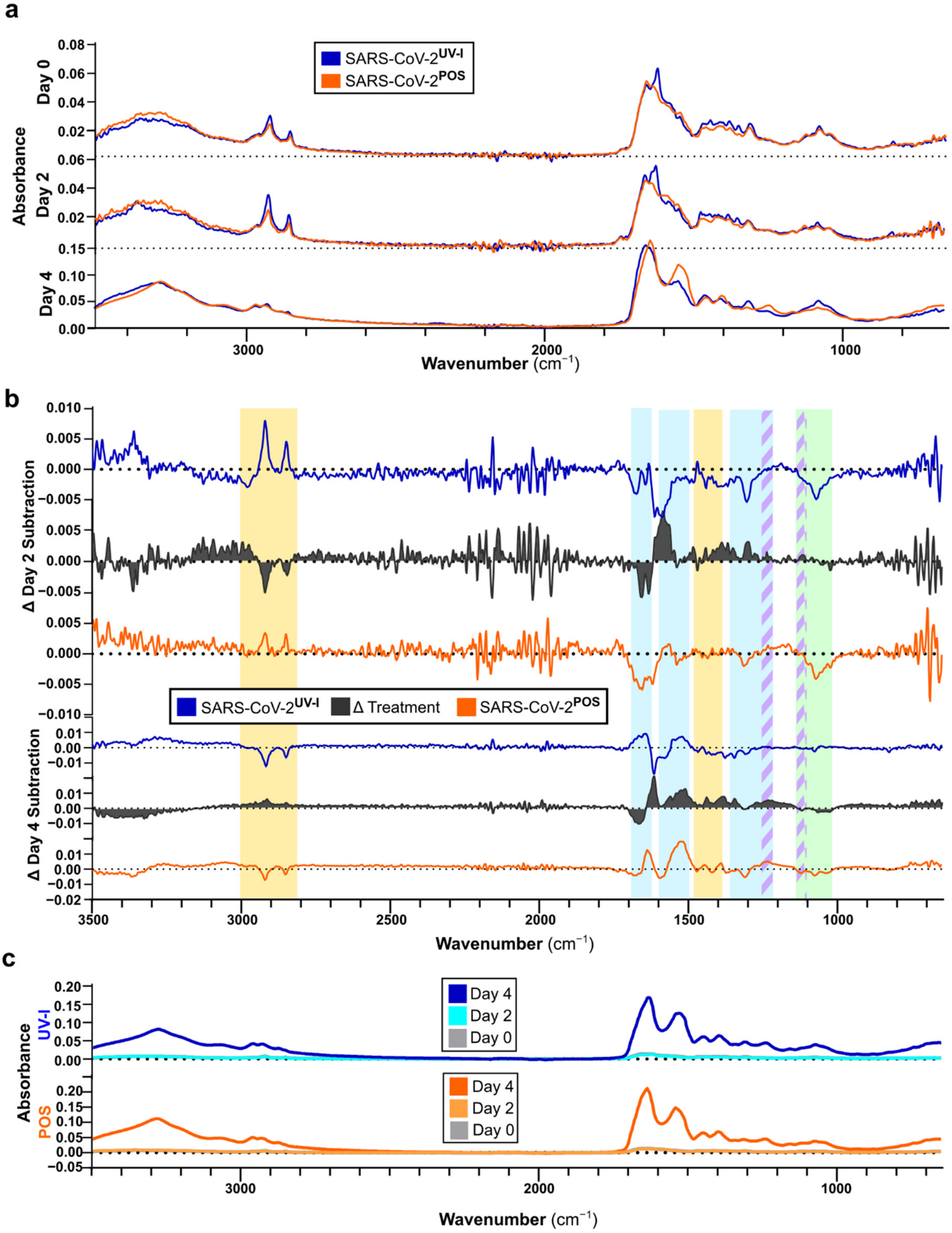

Figure S2. Full ATR-FTIR spectra and subtraction analysis of mouse oral lavage. a) ATR-FTIR spectra (3500-650 cm^{-1}) of SARS-CoV-2 (orange) and UV-inactivated SARS-CoV-2 (blue) groups per day 0 (baseline), day 2, and day 4. **b)** Spectra subtraction at days 2 and 4 from day 0 per each group. The 'Δ Treatment' (grey) condition is the difference between SARS-CoV-2^{POS} and SARS-CoV-$2^{UV\text{-}I}$ time-point subtractions as respectively illustrated. Bands signified by colors: Aliphatic (yellow), Amide I/II/III (cyan), Saccharide (green), phosphodiester asymmetric stretching ($\nu_{as}PO_2^-$) and symmetric stretching ($\nu_sPO_2^-$)

(purple stripes). **c)** Un-normalized spectra showing overlapping spectra for days 0, 2, and 4, for each respective group: Inactivated SARS-CoV-2 (top), SARS-CoV-2 (bottom).

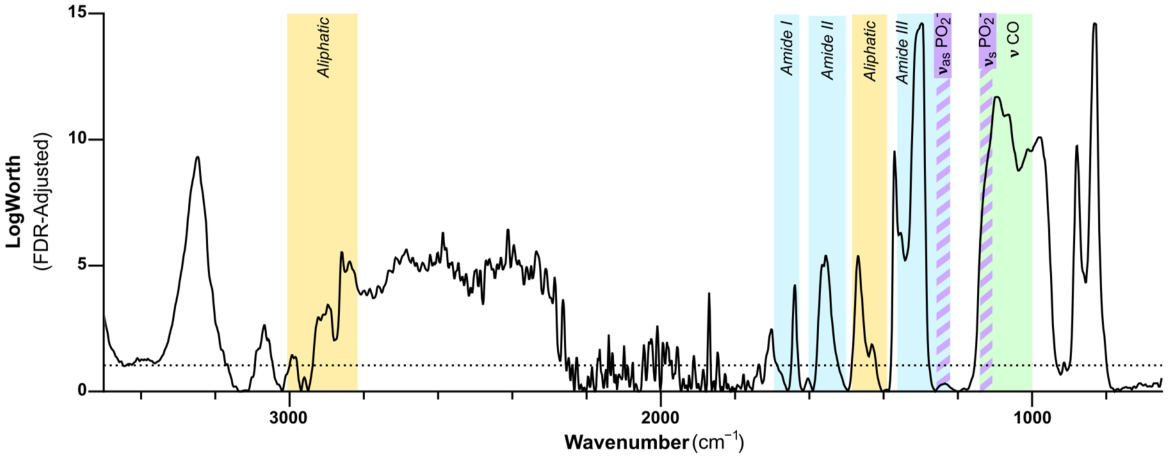

Figure S3. Significant features of in vivo infection mouse model using LogWorth FDR analysis. All bands of interest demonstrated significant changes by day 4; dotted line indicating FDR=1.3 (P<0.05). Features of aliphatic signals, protein, nucleic acid (RNA), and saccharides were all found to be significantly alterations by SARS-CoV-2 infection.

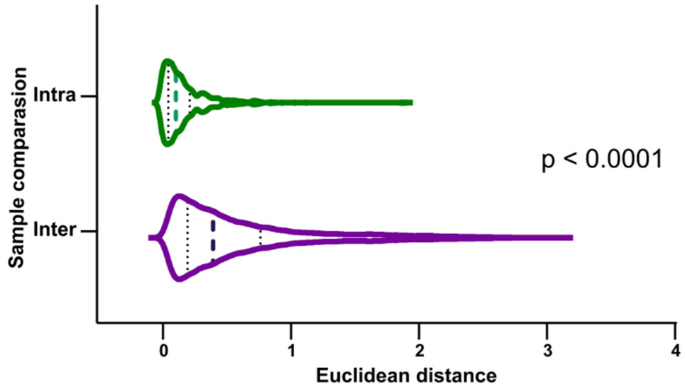

	Inter	Intra
Number of values	89,253	1,970
Minimum	0.0271	0.009915
25% Percentile	0.2233	0.07259
Median	0.4231	0.1333
75% Percentile	0.7929	0.2423
Maximum	3.126	1.92
Range	3.099	1.91
Mean	**0.6089**	**0.1925**
Std. Deviation	0.544	0.1941
Std. Error of Mean	0.001821	0.004373

Figure S4. Acceptable technical variance between replicates using pairwise Euclidean distancing analysis of human samples. Analysis comparing normalized FTIR spectra variance of technical replicates (Intra) to that of biological (Inter). A statistical difference of $p < 0.0001$ was observed when comparing distributions of the two groups.

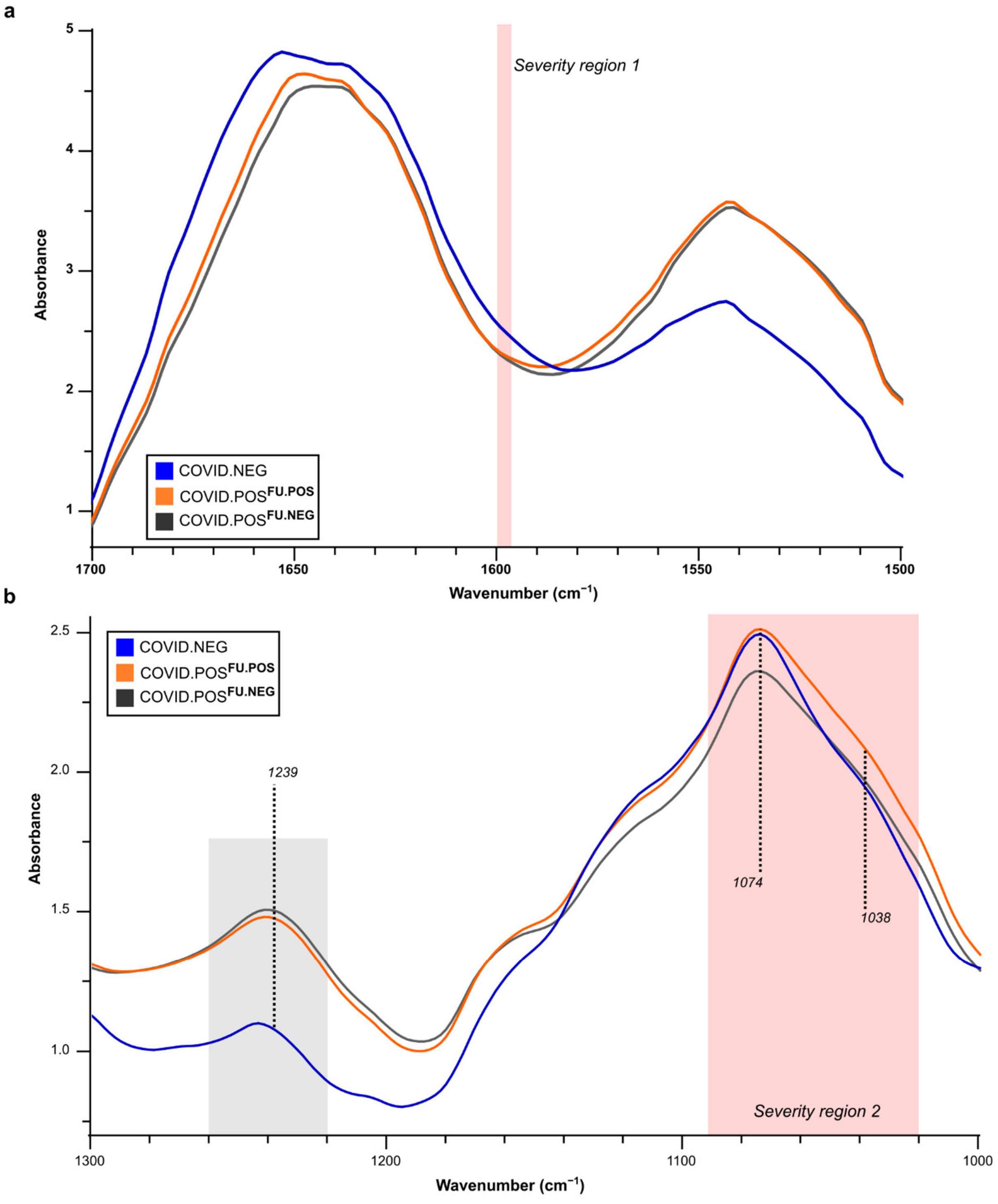

Figure S5. Human subject ATR-FTIR close-up of averaged spectra for three groups. a) Magnified view of the averaged spectra of the three groups along the range 1700-1500 cm^{-1}. Groups are identified as COVID.NEG (blue), COVID.POS$^{FU.POS}$ (orange) and COVID.POS$^{FU.NEG}$ (black). b) Magnified view along the range, 1300-1000 cm^{-1}. Wavenumbers labelled as 1239, 1074, and 1038 cm^{-1}, have been shown to represent SARS-CoV-2 RNA, while 1260-1220 cm^{-1} (grey) also represents protein amyloid aggregates.[2]

Table S1. Summary of cohorts

	**i) QIMRB/TPCH**	**ii) TPCH**	**iii) NSW HP**
Healthy	42/2	0	0
COVID.POS			
Saliva PCR positive	0	6	40
Saliva PCR negative	0	4	10
**Total (n)**	**44**	**10**	**50**

TPCH, The Prince Charles Hospital; QIMRB, QIMR Berghofer Medical Research Institute; NSWHP, New South Wales Health Pathology
